# Prediction of progressive pulmonary fibrosis in patients with anti-synthetase syndrome-associated interstitial lung disease

**DOI:** 10.1007/s10067-023-06570-3

**Published:** 2023-03-17

**Authors:** Hongyan Fu, Ziyao Zheng, Zhenping Zhang, Yanjuan Yang, Jieda Cui, Zhaojun Wang, Jing Xue, Shuhong Chi, Mengshu Cao, Juan Chen

**Affiliations:** 1 Department of Key Laboratory of Ningxia Stem Cell and Regenerative Medicine, Institute of Medical Sciences, General Hospital of Ningxia Medical University, Yinchuan, 750004 Ningxia China; 2 Department of Pulmonary and Critical Care Medicine, General Hospital of Ningxia Medical University, Yinchuan, 750004 Ningxia China; 3 Ningxia Medical University, Yinchuan, 750004 Ningxia China; 4 Department of Radiology, General Hospital of Ningxia Medical University, Yinchuan, 750004 China; 5 Department of Pulmonary and Critical Care Medicine, General Hospital of Xinmi Traditional Chinese Medicine, Xinmi, 452370 Henan China; 6 Institute of Human Stem Cell Research, General Hospital of Ningxia Medical University, Yinchuan, 750004 Ningxia China; 7 Department of Rheumatology, General Hospital of Ningxia Medical University, Yinchuan, 750004 Ningxia China; 8 Department of Respiratory and Critical Care Medicine, The Affiliated Drum Tower Hospital of Nanjing University Medical School, No. 321 Zhongshan Road, Nanjing, 210008 Jiangsu China; 9 Department of Critical Care Medicine, General Hospital of Ningxia Medical University, Yinchuan, 750004 Ningxia China

**Keywords:** Anti-synthetase syndrome, Interstitial lung disease, Progressive pulmonary fibrosis, Risk factors

## Abstract

**Objective:**

Interstitial lung disease (ILD) is a common extramuscular manifestation of the anti-synthetase syndrome (ASS). Patients with ASS-ILD are at risk in developing a progressive fibrosing phenotype despite appropriate treatments. This study investigated the risk factors and the predictive value of multiple risk factors for progressive pulmonary fibrosis (PPF) in patients with ASS-ILD.

**Methods:**

Ninety patients with a diagnosis of ASS and evidence of ILD on high-resolution computed tomography (HRCT) were recruited. Among them, 72 participants completed follow-up for more than 12 months. These patients were further divided into a PPF-ASS group (*n* = 18) and a non-PPF-ASS group (*n* = 54). Logistic regression analysis was performed to investigate the risk factors for PPF. The predictive value of the combined risk factors for predicting PPF were analyzed by a ROC curve.

**Results:**

The PPF-ASS group had a higher rate of positive non-Jo-1 antibodies, a significantly higher neutrophil-to-lymphocyte ratio (NLR) and serum lactate dehydrogenase (LDH), and a significantly lower PaO_2_/FiO_2_ ratio and diffusing capacity for carbon monoxide (DLCO%pred) than the non-PPF-ASS group. In addition, elevated serum Krebs von den Lungen-6 (KL-6) level and reticular opacities were significantly more common, and corticosteroid monotherapy at onset was administered more frequently in the PPF-ASS group. The median duration of follow-up was 37.4 months, survival was poorer in the PPF-ASS group, and the overall survival was 88.9%. Multivariate regression analysis further revealed that positive non-Jo-1 antibodies, NLR, and KL-6 were independent risk factors for PPF. These combined indexes had good accuracy (area under the curve = 0.874) in predicting PPF in patients with ASS-ILD.

**Conclusion:**

Positive non-Jo-1 antibodies, NLR, and serum KL-6 are independent risk factors for PPF in patients with ASS-ILD. Monitoring these markers can potentially predict PPF in this group of patients.

**Table:** 

**Key Points**
*• Positive non-Jo-1 antibodies, NLR, and serum KL-6 are independent risk factors associated with PPF in patients with ASS-ILD.*
*• Monitoring non-Jo-1 antibodies, NLR, and serum KL-6 can potentially predict PPF in patients with ASS-ILD.*

**Supplementary Information:**

The online version contains supplementary material available at 10.1007/s10067-023-06570-3.

## Introduction

Anti-synthetase syndrome (ASS) is a subgroup of idiopathic inflammatory myopathies (IIMs), which is strongly associated with myositis, mechanic’s hands, fever and/or Raynaud’s phenomenon, interstitial lung disease (ILD), and positive anti-aminoacyl-tRNA-synthetase antibodies (ARSs) [1, 2]. ARSs are enzymes responsible for the synthesis of aminoacyl-tRNAs, including anti-histidyl (anti-Jo-1), anti-glycyl (anti-EJ), anti-alanyl (anti-PL-7), anti-threonyl (anti-PL-12), anti-asparaginyl (anti-KS), anti-isoleucyl (anti-OJ), anti-phenylalanyl (anti-Zo), and anti-threonyl (anti-Ha) [3]. Anti-Jo-1 is the most common ARS subtype and is associated with a high incidence of classical myositis, whereas anti-PL-7, anti-PL-12, and anti-EJ are associated with a high incidence of ILD, and the remaining ARSs (anti-KS, anti-OJ, anti-Zo, and anti-Ha) are rarely found in < 2% the patients with IIM [4]. ILD is a major determinant of morbidity and mortality in patients with ASS, and several studies suggest that different ARS subtypes could be related to mortality in ASS patients [5–7].

Progressive pulmonary fibrosis (PPF), also known as progressive fibrosing ILD (PF-ILD), is the continuous worsening of any pre-existing ILD other than idiopathic pulmonary fibrosis (IPF) and is characterized by the deterioration of respiratory symptoms and physiological or radiological evidence of disease progression [8, 9]. The PERSEIDS study involving six European countries reported that the estimated incidence and prevalence of non-IPF PF-ILD were 2.1–14.5/10^5^ person-years and 6.9–78.0/10^5^ persons, respectively [10]. A large study conducted by pulmonologists, rheumatologists, and internists from multiple countries found that 18–32% of patients with non-IPF ILD had signs of disease progression and fibrosis, and the time from symptom onset to death was 61–80 months [11]. Patients with IIM-ILD are also at risk of developing a progressive fibrosing phenotype despite standard treatments that are performed [8]. However, the proportion of patients with the progressive fibrosing phenotype varies by ILD subtypes, and the incidence in patients with ASS-ILD is unknown [12]. Biomarkers can be useful for the clinical prediction and monitoring of patients with ILD with a progressive fibrosing phenotype [13]. Krebs von den Lungen-6 (KL-6) is a glycoprotein expressed in type II pneumocytes and bronchiolar epithelial cells, which promotes the proliferation, migration, and survival of lung fibroblasts [14]. Serum KL-6 concentrations are elevated in patients with ILDs, including connective tissue disease (CTD), and serum KL-6 is useful for diagnosis, prognosis, and treatment response monitoring [15]. Moreover, in patients with PPF, biomarkers can help predict prognosis and response to therapy, and monitor treatment response. To date, no biomarker has been validated in these patients, and the early recognition of patients with ASS-ILD who are at risk of developing PPF remains challenging. Thus, this study investigated the risk factors for PPF and assessed the predictive value of the combination of risk factors for PPF in patients with ASS-ILD.

## Methods

### Ethics approval

This study was approved by the Human Research Ethics Committee of the General Hospital of Ningxia Medical University (Protocol No. 2019-381). Written consent was obtained from all patients.

### Study population

This retrospective single-center study was conducted at the General Hospital of Ningxia Medical University (Yinchuan, China). Patients diagnosed with ASS-ILD hospitalized from October 2017 to June 2020 were enrolled after a multidisciplinary evaluation (pulmonologists, radiologists, and rheumatologists).

**Fig. 1:**
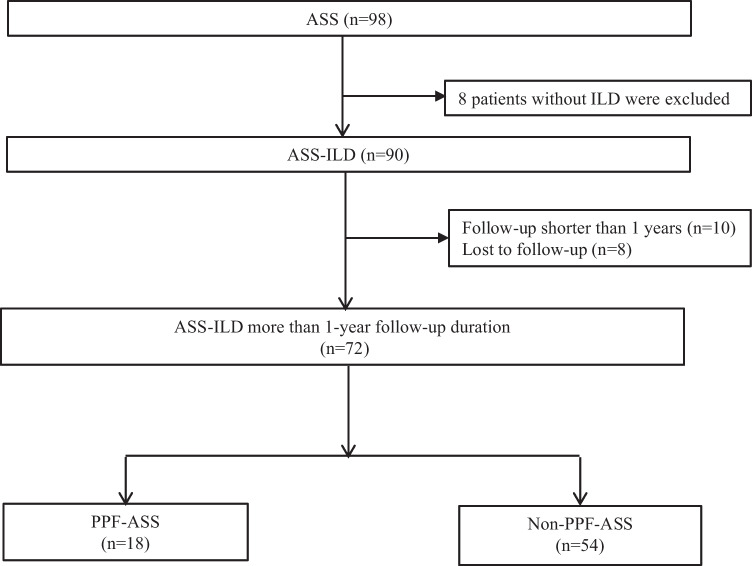
Study flow chart. ASS-ILD: anti-synthetase syndrome-associated interstitial lung disease; PPF: progressive pulmonary fibrosis

In total, 90 patients diagnosed with ASS-ILD were enrolled. Of these, 72 participants were included in the analysis, and 18 were excluded because of follow-up of less than 1 year (10 cases) and loss to follow-up (8 cases) (Fig. 1).

The criteria for patients included were as follows: (a) patients with polymyositis (PM)/dermatomyositis (DM) based on the 1975 Bohan and Peter’s criteria, and only definite PM/DM patients were included [16, 17]; (b) patients with ASS based on the criteria proposed by Solomon et al. [18]; and (c) patients with ILD based on the 2013 American Thoracic Society (ATS)/European Respiratory Society (ERS) criteria for idiopathic interstitial pneumonia (IIP) [19].

### Data collection and samples

Clinical and survival data were obtained from hospital records or telephone follow-up. At the baseline, data on general characteristics, diagnosis, pulmonary function test (PFT), laboratory variables, radiological findings, and pharmacologic treatments were collected. Respiratory symptoms, HRCT findings, and pulmonary function test (PFT) results were evaluated at hospital visit every 3–6 months, and diagnosis and follow-up data were discussed by a multidisciplinary team. The follow-up time was defined as the date from the initial diagnosis (October 2017) to the end of the study (August 2022). Survival time was defined as the date of diagnosis to the date of death or the date of the last follow-up. Blood samples were collected from every patient during the initial diagnosis of ASS-ILD at our hospital and stored at − 80 °C for further analysis.

### Detection of ARS subtypes

ARS subtypes were identified on admission using a line blot immunoassay (Oumeng Medical Laboratory Diagnosis Co., Hangzhou, China) according to the manufacturer’s instructions. An intensity level of 2 + or more was deemed positive [20]. The ARS subtypes evaluated in this study were anti-Jo-1 antibodies (29 patients) and non-Jo-1 antibodies (anti-PL-7 (19 patients), anti-PL-12 (11 patients), and anti-EJ (13 patients)).

### Serum levels of GDF-15, KL-6, and Wnt-5a

Serum levels of GDF-15, KL-6, and Wnt-5a were measured by enzyme-linked immunosorbent assays (Elabscience Biotechnology Co. Ltd, China) according to the manufacturer’s instructions.

### HRCT features

HRCT scans comprised 1.0–1.5-mm collimation sections (window level, 600 Hounsfield units; window width, 1500 Hounsfield units). Images were interpreted independently by two experienced thoracic radiologists with expertise in ILD who were blinded to the clinical information. They independently reviewed the HRCT scans by visual assessment and discrepancies were adjudicated by consensus. HRCT characteristics, including ground-glass opacities (GGO), consolidation, irregular linear opacities, reticular opacities, bronchovascular bundle thickening, traction bronchiectasis and bronchiolectasis, and honeycombing, were reported according to the Fleischner Society definitions [21]. The HRCT patterns of ILD were classified according to the 2013 ATS/ERS criteria for IIP, which were classified as usual interstitial pneumonia (UIP), nonspecific interstitial pneumonia (NSIP), organizing pneumonia (OP), NSIP/OP overlap, and unclassifiable [19]. The extent of pulmonary fibrosis was assessed visually by side-by-side comparison.

### Definition of PPF

PPF was defined according to the 2022 Official Clinical Practice Guideline as a combination of at least two of the following three domains within 12 months: worsening respiratory symptoms, physiological evidence of disease progression (absolute decline in FVC > 5% predicted and or DLCO (corrected for Hb) > 10% predicted), or radiological evidence of disease progression (increased extent of fibrotic features) [9].

### Statistical analysis

Categorical variables were presented as frequencies, and differences between groups were estimated using the chi-square test or Fisher’s exact test. Differences in continuous variables were determined using an unpaired *t* test or Mann–Whitney *U* test. The risk factors of PPF were analyzed using univariate logistic regression analysis, and the independent risk factors of PPF were analyzed using multivariate logistic regression analysis. The predictive value of the best combination of predictive factors was identified using receiver operating characteristic (ROC) curves. The cumulative survival rate was calculated using the Kaplan–Meier method. A *p* value of less than 0.05 was considered statistically significant.

## Results

### Patient characteristics and ARS subtypes

The clinical characteristics and ARS subtypes of the patients with ASS-ILD are summarized in Table 1. Seventy-two patients (32 (44.4%) with PM and 40 (55.6%) with DM) were included in the analysis. The mean age at disease onset was 56.6 ± 12.6 years, and 51 patients (70.8%) were female. Among ARS subtypes, the frequency of positive anti-Jo-1 antibodies was 40.3%, and the frequency of positive anti-PL-7, anti-PL-12, and anti-EJ antibodies (non-Jo-1 antibodies) were 26.4%, 15.3%, and 18.1%, respectively.

**Table 1:** Clinical characteristics and ARS subtypes between the PPF-ASS and non-PPF-ASS groups

	Total (*n* = 72)	PPF-ASS (*n* = 18)	Non-PPF-ASS (*n* = 54)	χ^2^/*t*	*p*
General characteristics					
Age at diagnosis, years	56.6 ± 12.6	60.3 ± 14.6	55.4 ± 11.7	1.428	0.158
Sex, female	51 (70.8)	10 (55.6)	41 (75.9)	2.711	0.100
Organ manifestations					
Fever	26 (36.1)	9 (50.0)	17 (31.5)	2.007	0.157
Myositis	53 (73.6)	15 (83.3)	38 (70.4)	1.168	0.280
Arthritis/arthralgia	31 (43.1)	7 (38.9)	24 (44.4)	0.170	0.680
Muscle weakness	45 (62.5)	12 (66.7)	33 (61.1)	0.178	0.673
Raynaud phenomenon	24 (33.3)	7 (38.9)	17 (31.5)	0.333	0.564
Mechanic’s hand	42 (58.3)	7 (38.9)	35 (64.8)	3.733	0.053
Heliotrope rash	23 (31.9)	7 (38.9)	16 (29.6)	0.532	0.466
Shawl sign	10 (13.9)	4 (22.2)	6 (11.1)	N/A	0.255
Gottron papules	14 (19.4)	2 (11.1)	12 (22.2)	N/A	0.494
Gottron sign	26 (36.1)	4 (22.2)	22 (40.7)	N/A	0.257
Diagnosis					
PM	32 (44.4)	11 (61.1)	21 (38.9)	2.700	0.100
DM	40 (55.6)	7 (38.9)	33 (61.1)		
ARS subtypes					
Positive Anti-Jo-1 antibody	29 (40.3)	3 (16.7)	26 (48.1)	N/A	0.026*
Positive non-Jo-1 antibodies	43 (59.7)	15 (83.3)	28 (51.9)		
Positive anti-PL-7 antibody	19 (26.4)	6 (33.3)	13 (24.1)	0.596	0.440
Positive anti-PL-12 antibody	11 (15.3)	3 (16.7)	8 (14.8)	N/A	1.000
Positive anti-EJ antibody	13 (18.1)	6 (33.3)	7 (13.0)	3.786	0.052

Data are presented as the mean ± standard deviation or *n* (%)

*Anti-EJ* anti-glycyl, *anti-Jo-1* anti-histidyl, *anti-PL-7* anti-alanyl, *anti-PL-12* anti-threonyl, *ARS* anti-aminoacyl-tRNA-synthetase, *ASS* anti-synthetase syndrome, *DM* dermatomyositis, *N/A* not applicable, *PPF* progressive pulmonary fibrosis, *PM* polymyositis

^*^p < 0.05

Eighteen (25.0%) and 54 (75.0%) ASS-ILD patients were classified as PPF (PPF-ASS group) and non-PPF (non-PPF-ASS group), respectively. The clinical characteristics of patients in these two groups are shown in Table 1. The PPF-ASS group had a higher rate of positive non-anti-Jo-1 antibodies (*p* = 0.026).

### Laboratory findings and PFT results

The laboratory findings and PFT results of patients in the PPF-ASS and non-PPF-ASS groups are shown in Table 2. The neutrophil-to-lymphocyte ratio (NLR) and lactic dehydrogenase (LDH) were significantly higher in the PPF-ASS group than that of the non-PPF-ASS group (*p* = 0.001 and *p* = 0.020, respectively). In addition, PaO_2_/FiO_2_ ratio and baseline diffusing capacity for carbon monoxide (DLCO%pred) were significantly lower in the PPF-ASS group than those in the non-PPF-ASS group (*p* = 0.041 and *p* = 0.037, respectively).

**Table 2:** Laboratory findings and PFT results between the PPF-ASS and non-PPF-ASS groups

	Total (*n* = 72)	PPF-ASS (*n* = 18)	Non-PPF-ASS (*n* = 54)	χ^2^/*t*	*p*
NLR	4.5 ± 3.5	6.8 ± 5.1	3.8 ± 2.4	N/A	0.001*
LMR	3.1 ± 1.8	3.1 ± 2.1	3.1 ± 1.7	N/A	0.594
PaO_2_/FiO_2_ ratio	310.5 ± 84.5	275.4 ± 83.6	322.2 ± 82.2	0.104	0.041*
LDH, U/L	597.2 ± 501.8	886.3 ± 736.8	500.8 ± 354.1	N/A	0.020*
CRP, mg/L	19.9 ± 41.8	39.6 ± 65.7	13.8 ± 28.0	N/A	0.059
ESR, mm/h	21.8 ± 23.7	29.2 ± 33.3	19.3 ± 19.3	N/A	0.298
ALT, U/L	39.8 ± 34.3	50.5 ± 36.5	36.2 ± 33.2	N/A	0.057
AST, U/L	31.3 ± 23.7	37.9 ± 24.7	29.1 ± 23.3	N/A	0.170
Creatine kinase, UI/L	414.9 ± 890.6	805.8 ± 1530.6	284.6 ± 491.8	N/A	0.224
PFT results at diagnosis, % predicted					
FVC	69.1 ± 12.8	65.2 ± 11.1	70.4 ± 13.2	− 1.498	0.139
DLCO	60.0 ± 14.7	53.8 ± 15.9	62.1 ± 13.8	− 2.126	0.037*

Data are presented as the mean ± standard deviation or *n* (%)

*ASS* anti-synthetase syndrome, *ALT* alanine aminotransferase, *AST* aspartate aminotransferase, *CRP* C-reactive protein, *DLCO* diffusing capacity for carbon monoxide, *FVC* forced vital capacity, *ESR* erythrocyte sedimentation rate, *LDH* lactate dehydrogenase, *LMR* lymphocyte-to-monocyte ratio, *N/A* not applicable, *NLR* neutrophil-to-lymphocyte ratio, *PFT* pulmonary function test, *PPF* progressive pulmonary fibrosis

^*^p < 0.05

### Serum biomarkers

The serum levels of GDF-15, KL-6, and Wnt-5a in the PPF-ASS and non-PPF-ASS groups are shown in Table 3. Serum KL-6 was higher in the PPF-ASS group (1009.3 ± 382.0 U/mL) compared to the non-PPF-ASS group (758.0 ± 241.7 U/mL) (*p* = 0.011). However, there was no significant difference in GDF-15 and Wnt-5a between these two groups.

**Table 3:** Serum biomarkers between the PPF-ASS and non-PPF-ASS groups

	Total (*n* = 72)	PPF-ASS (*n* = 18)	Non-PPF-ASS (*n* = 54)	χ^2^/*t*	*p*
KL-6, U/mL	820.9 ± 300.9	1009.3 ± 382.0	758.0 ± 241.7	N/A	0.011*
GDF-15, pg/mL	221.5 ± 91.7	226.8 ± 102.5	219.7 ± 88.8	0.408	0.778
Wnt-5a, ng/mL	3.1 ± 4.0	3.6 ± 4.7	3.0 ± 3.7	N/A	0.607

Data are presented as the mean ± SD or *n* (%)

*ASS* anti-synthetase syndrome, *GDF-15* growth differentiation factor 15, *KL-6* Krebs von den Lungen-6, *N/A* not applicable, *PPF* progressive pulmonary fibrosis, *Wnt* wingless

^*^p < 0.05

### HRCT findings

**Fig. 2:**
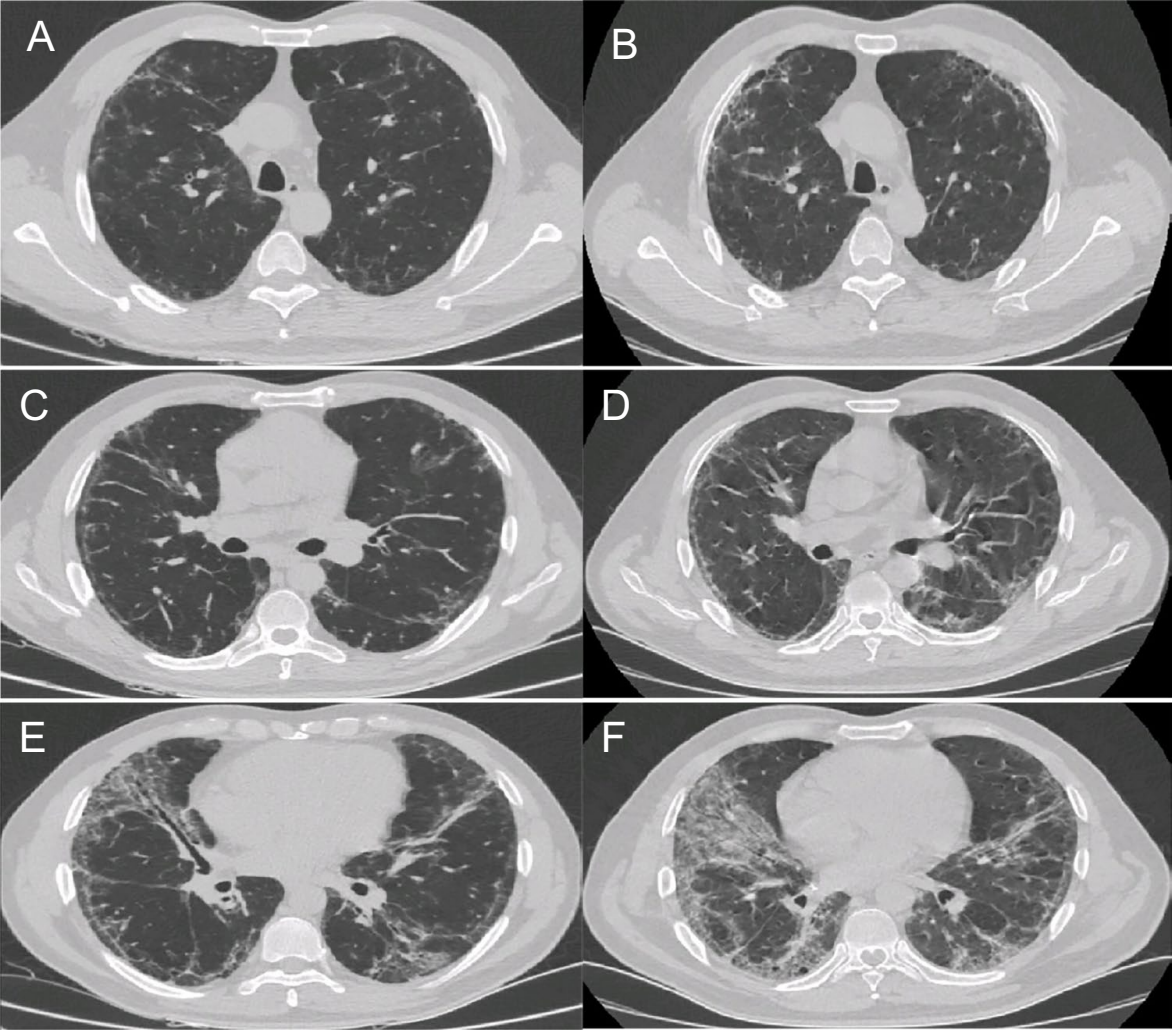
Progressive pulmonary fibrosis due to fibrotic nonspecific interstitial pneumonia (NSIP). **A**, **C**, **E** A 49-year-old male with anti-EJ antibody-positive dermatomyositis and interstitial lung disease showing reticular opacities, ground-glass opacities (GGOs), and traction bronchiectasis in the peribronchovascular and subpleural region of the lower lung, typical of fibrotic NSIP. **B**, **D**, **F** There was progressive fibrosis associated with reticular opacities, GGOs with traction bronchiectasis, and increased lobar volume loss at the 1-year follow-up

**Fig. 3:**
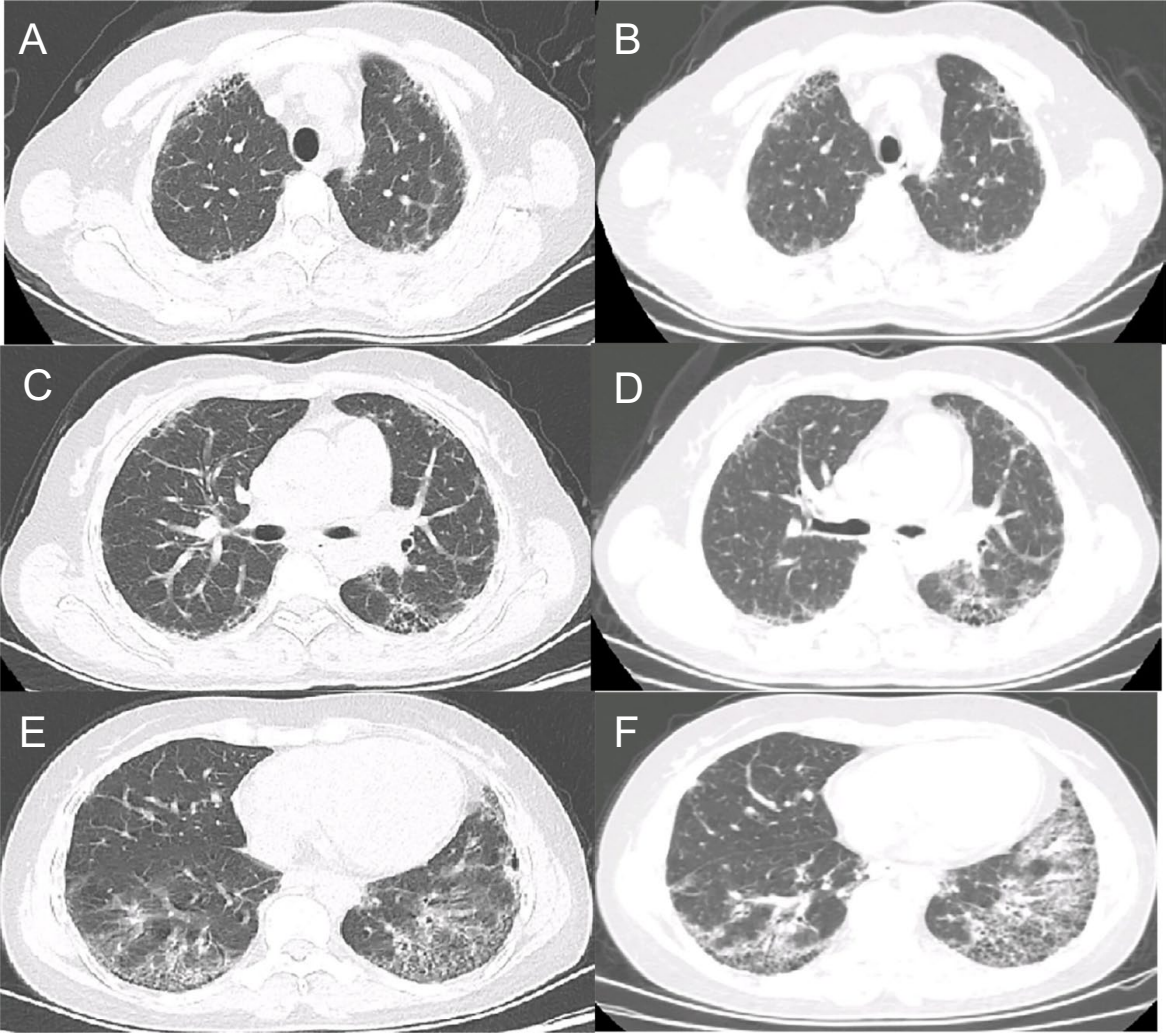
Progressive pulmonary fibrosis due to probable usual interstitial pneumonia. A 32-year-old female with anti-Jo-1 antibody-positive dermatomyositis/interstitial lung disease. **A**, **C**, **E** High-resolution computed tomography (HRCT) images showing moderate honeycombing and reticular abnormalities in the subpleural and basal regions. **B**, **D**, **F** HRCT images showing a substantial increase in the extent of reticular opacities and ground-glass opacities associated with traction bronchiectasis and increased lobar volume loss at the 1-year follow-up

**Fig. 4:**
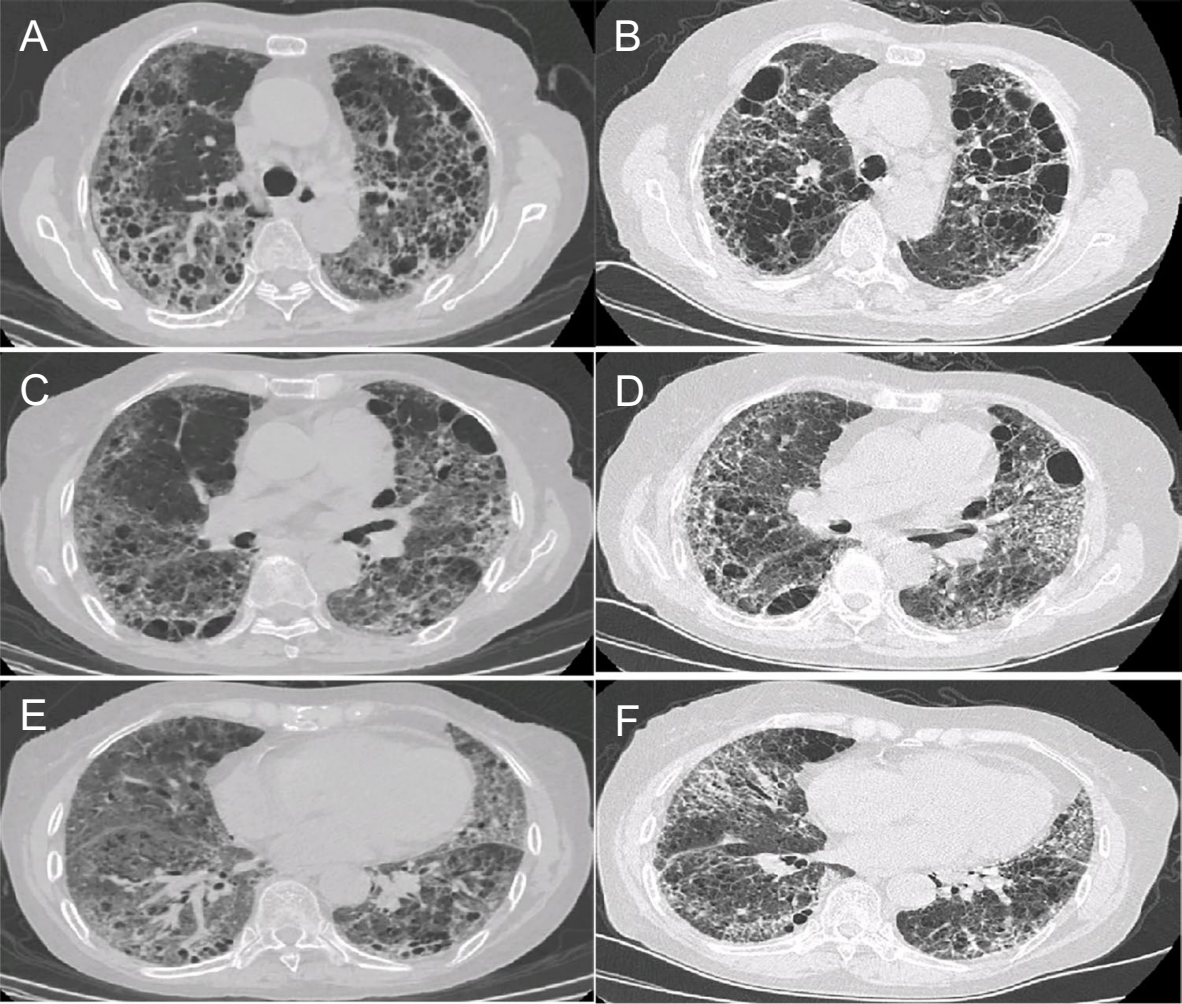
Progressive pulmonary fibrosis of unknown etiology. A 72-year-old female with anti-PL-12 antibody-positive dermatomyositis/interstitial lung disease. **A**, **C**, **E** High-resolution computed tomography (HRCT) images showing extensive honeycombing and cysts in the upper-middle lung and extensive ground-glass opacities and reticular abnormalities in the upper and lower lung. **B**, **D**, **F** HRCT images showing a substantial increase in the extent of reticular opacities and ground-glass opacities with traction bronchiectasis and increased lobar volume loss at the 7-month follow-up

**Fig. 5:**
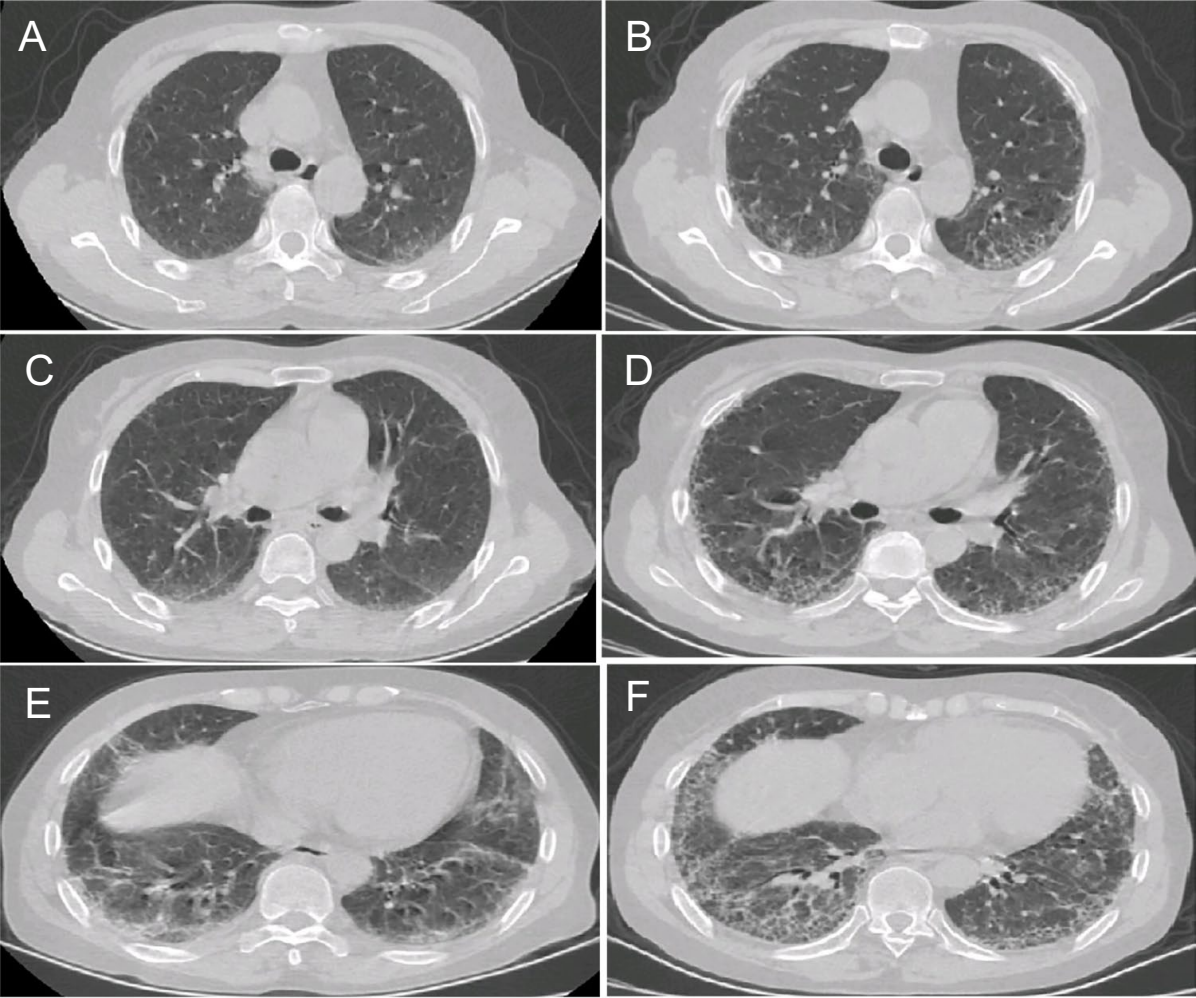
Progressive pulmonary fibrosis due to nonspecific interstitial pneumonia/organizing pneumonia overlap. A 64-year-old female with anti-PL-12 antibody-positive dermatomyositis/interstitial lung disease. **A**, **C**, **E** High-resolution computed tomography (HRCT) images showing consolidations superimposed on a background of ground-glass opacities in the subpleural region of the lower lung. **B**, **D**, **F** HRCT images showing new reticular opacities and traction bronchiectasis at the 7-month follow-up

HRCT findings are summarized in Table 4. The most common HRCT pattern was NSIP (41.7%), followed by NSIP/OP overlap (37.5%), OP (15.3%), UIP (4.2%), and unclassifiable (1.4%). The PPF-ASS group was more likely to have reticular opacities (*p* = 0.014). Representative HRCT images of PPF are shown in Figs. 2, 3, 4, and 5.

**Table 4:** HRCT findings between the PPF-ASS and non-PPF-ASS groups

	Total (*n* = 72)	PPF-ASS (*n* = 18)	Non-PPF-ASS (*n* = 54)	χ^2^/*t*	*p*
HRCT features					
Ground-glass opacity	67 (93.1)	18 (100.0)	49 (90.7)	1.791	0.181
Consolidation	39 (54.2)	7 (39.9)	32 (59.3)	2.256	0.133
Irregular linear opacities	29 (40.3)	6 (33.3)	23 (42.6)	0.481	0.488
Reticular opacities	38 (52.8)	14 (77.8)	24 (44.4)	6.019	0.014*
Traction bronchiectasis and bronchiolectasis	40 (55.6)	12 (66.7)	28 (51.9)	1.200	0.273
Bronchovascular bundle thickening	28 (38.9)	7 (38.9)	21 (38.9)	0.000	1.000
Honeycombing	3 (4.2)	2 (11.1)	1 (1.9)	N/A	0.152
HRCT pattern, *n* (%)					
NSIP	30 (41.7)	9 (50.0)	21 (38.9)	0.686	0.408
OP	11 (15.3)	0 (0.0)	11 (20.4)	N/A	0.055
NSIP with OP overlap	27 (37.5)	6 (27.8)	21 (38.9)	0.178	0.673
UIP	3 (4.2)	2 (11.1)	1 (1.9)	N/A	0.152
Unclassifiable	1 (1.4)	1 (5.6)	0 (0.0)	N/A	0.250

Data are presented as the mean ± standard deviation or *n* (%)

*ASS* anti-synthetase syndrome, *HRCT* high-resolution computed tomography, *N/A* not applicable, *NSIP* nonspecific interstitial pneumonia, *OP* organizing pneumonia, *PPF* progressive pulmonary fibrosis, *UIP* usual interstitial pneumonia

^*^p < 0.05

### Treatment and outcomes

**Fig. 6:**
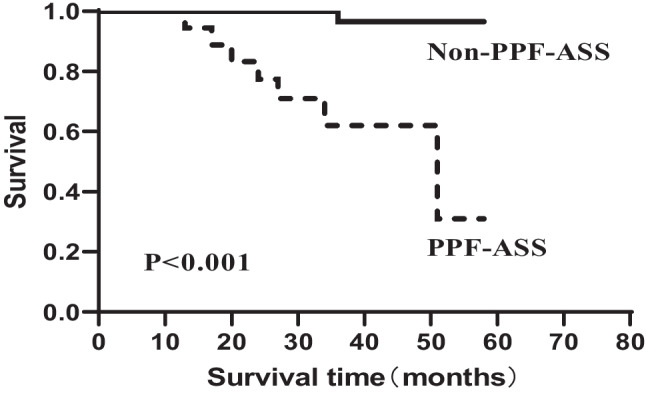
Comparison of survival curves between the PPF-ASS and non-PPF-ASS groups among patients with ASS-ILD. Survival rates were calculated using the Kaplan–Meier method and were compared using the log-rank test. Solid line: ASS-ILD patients without progressive pulmonary fibrosis (PPF). Dashed line: ASS-ILD patients with PPF

Treatment and outcomes are presented in Table 5. All patients received corticosteroids (CS) alone or combined with other therapies at onset. The frequency of CS monotherapy at onset was significantly higher in the PPF-ASS group (*p* = 0.009). The median duration of follow-up was 37.4 months; 64 (88.9%) patients survived, including 11 (61.1%) in the PPF-ASS group and 53 (98.1%) in the non-PPF-ASS group. However, the survival was poorer in the PPF-ASS group relative to the non-PPF-ASS group (*p* < 0.001), as shown in Kaplan–Meier survival curves in Fig. 6.

**Table 5:** Treatment and outcome between the PPF-ASS and non-PPF-ASS groups

	Total (*n* = 72)	PPF-ASS (*n* = 18)	Non-PPF-ASS (*n* = 54)	χ^2^/*t*	*p*
Treatment, *n* (%)					
CS monotherapy	19 (26.4)	9 (50.0)	10 (18.5)	6.888	0.009*
CS + CYC	19 (26.4)	4 (22.2)	15 (27.8)	N/A	0.214
CS + CNI	16 (22.2)	2 (11.1)	14 (25.9)	N/A	0.326
CS + HCQ	6 (8.3)	0 (0.0)	6 (11.1)	N/A	0.326
CS + AZA	2 (2.8)	0 (0.0)	2 (3.7)	N/A	1.000
CS + MMF	1 (1.4)	0 (0.0)	1 (1.9)	N/A	1.000
CS + CYC + IG	7 (9.7)	2 (11.1)	5 (9.3)	N/A	1.000
CS + CYC + CNI	1 (1.4)	1 (5.6)	0 (0.0)	N/A	0.250
CS + CNI + IG	1 (1.4)	0 (0.0)	1 (1.9)	N/A	1.000
Outcome, *n* (%)					
Survivals	64 (88.9)	11 (61.1)	53 (98.1)	N/A	< 0.001*

Data are presented as the mean ± SD or *n* (%)

*ASS* anti-synthetase syndrome, *AZA* azathioprine, *CYC* cyclophosphamide, *CNI* calcineurin inhibitor, *CS* corticosteroids, *HCQ* hydroxychloroquine, *IG* immunoglobulin, *MMF* mycophenolate mofetil, *N/A* not applicable, *PPF* progressive pulmonary fibrosis

^*^p < 0.05

### Predictive factors for PPF during long-term follow-up in ASS-ILD

The factors contributing to PPF during long-term follow-up in patients with ASS-ILD were analyzed. The results of univariate and multivariate logistic regression analysis are presented in Table 6.

**Table 6:** Predictive factors for PPF in ASS-ILD (univariate and multivariate logistic regression analysis)

	OR	95% CI	*p*
Univariate logistic regression analysis			
Positive non-Jo-1 antibodies	4.643	1.204–17.903	0.026*
PaO_2_/FiO_2_ ratio	2.320	0.760–7.085	0.140
NLR	5.20	1.652–16.369	0.005*
LDH (U/L)	3.143	1.042–9.477	0.042*
DLCO, % predicted	3.500	1.136–10.779	0.029*
KL-6 (U/mL)	14.655	1.819–118.074	0.012*
Reticular opacities	4.375	1.274–15.029	0.019*
CS monotherapy	4.4	1.392–13.912	0.012*
Multivariate logistic regression analysis			
Positive non-Jo-1 antibodies	8.88	1.243–63.462	0.030*
NLR	6.412	1.166–35.255	0.033*
KL-6 (U/mL)	27.796	1.978–390.596	0.014*

*ASS-ILD* anti-synthetase syndrome-associated interstitial lung disease, *anti-Jo-1* anti-histidyl, *CS* corticosteroids, *DLCO* diffusing capacity for carbon monoxide, *KL-6* Krebs von den Lungen-6, *LDH* lactate dehydrogenase, *NLR* neutrophil-to-lymphocyte ratio, *PPF* progressive pulmonary fibrosis

^*^p < 0.05

Univariate analysis revealed that positive non-Jo-1 antibodies (odds ratio (OR): 4.643, 95% confidence interval (CI): 1.204–17.903, *p* = 0.026), NLR (OR: 5.20, 95% CI: 1.652–16.369, *p* = 0.005), serum LDH (OR: 3.143, 95% CI: 1.042–9.477, *p* = 0.042), DLCO%pred (OR: 3.5, 95% CI: 1.136–10.779, *p* = 0.029), serum KL-6 (OR: 14.655, 95% CI: 1.819–118.074, *p* = 0.012), reticular opacities (OR: 4.375, 95% CI: 1.274–15.029, *p* = 0.019), and CS monotherapy (OR: 4.4, 95% CI: 1.392–13.912, *p* = 0.012) were risk factors for PPF in patients with ASS-ILD. Multivariate analysis revealed that positive non-Jo-1 antibodies (OR: 8.88, 95% CI: 1.243–63.462, *p* = 0.030), NLR (OR: 6.412, 95% CI: 1.166–35.255, *p* = 0.033), and serum KL-6 (OR: 27.796, 95% CI: 1.978–390.596, *p* = 0.014) were independent risk factors for PPF in patients with ASS-ILD.

### ROC analysis of the predictive ability of isolated and combined factors

In this study, the clinical factors for predicting PPF were positive non-Jo-1 antibodies, NLR, and serum KL-6, and the cutoff value for NLR and serum KL-6 values that best predicted PPF in patients with ASS-ILD were determined by ROC analysis. The optimal cutoff value for NLR was 4.05 (sensitivity: 72.2%, specificity: 74.1%), and the area under the curve (AUC) for NLR was 0.758 (Supplementary Table S1 and Supplementary Fig. S1). The optimal cutoff value for serum KL-6 was 644.71 U/mL (sensitivity: 94.4%, specificity: 46.3%), and the AUC for serum KL-6 was 0.702 (Supplementary Table S1 and Supplementary Fig. S2). Furthermore, the ROC analysis was conducted to reveal the best combination of clinical factors, and the results are shown in Supplementary Table S2 and Supplementary Fig. S3. When these clinical factors were combined, the predictive value of positive non-Jo-1 antibodies and NLR, combined with serum KL-6, was the highest (AUC = 0.874), and sensitivity and specificity were 94.4% and 64.8%, respectively.

## Discussion

ILD is a common extramuscular manifestation of ASS [22]. A small proportion of patients with ASS-ILD can present with PPF over the study period which is characterized by worsening respiratory symptoms, limited response to therapy, decreased quality of life, and potentially early death [8, 23]. Currently, there are limited data regarding the frequency and the predictive factors of PPF in patients with ASS-ILD available. This study aims to assess the risk factors for PPF and the predictive value of the combined factors for PPF in 72 patients with ASS-ILD. Based on the 2022 PPF definition criteria [9], our data showed that 25.0% of patients with ASS-ILD develop PPF during the follow-up period (median, 37.4 months), which was consistent with the previous literature [11]. Our results also provided the frequency of PPF in patients with ASS-ILD.

Biomarkers are useful for clinically evaluating and monitoring patients with ILD with a progressive fibrosing phenotype. KL-6 is a mucin protein strongly expressed on the surface of type II pneumocytes, regenerated during lung injuries in ILDs [24]. Honda et al. [25] observed that elevated KL-6 (mean, 802.4 U/mL) was associated with a higher frequency of lung fibrosis (traction bronchiectasis and architectural distortion) compared with normal KL-6 levels (mean, 305.7 U/mL). Therefore, elevated KL-6 may reflect fibrosis. A previous study reported that serum KL-6 had high sensitivity and specificity for diagnosing IIM-associated ILD and predicted ILD progression [26]. Moreover, serum KL-6 higher than 811 U/mL significantly predicted disease progression in ILD and IPF [27, 28]. Our results found that serum KL-6 was an independent risk factor for PPF in patients with ASS-ILD, which was up to 644.71 U/mL in patients with ASS-ILD. These findings indicate that serum KL-6 can be a predictive factor for long-term PPF in patients with ASS-ILD.

The clinical characteristics of patients with ARSs vary depending on the serological profile [29]. Aggarwal et al. [30] reported that ASS patients with positive non-Jo-1 antibody have worse survival compared with ASS patients with positive anti-Jo-1 antibody. Pulmonary fibrosis was the main cause of death in ASS patients. Marie et al. [31] showed that positive anti-PL-7/PL-12 ASS patients have a higher median score of fibrosis on HRCT than positive anti-Jo-1 ASS patients at the last follow-up (median, 34 months), and positive anti-PL-7/PL-12 ASS patients have a poor outcome despite therapies. In our study, the PPF-ASS group had a higher rate of positive non-Jo-1 antibodies than that of the non-PPF-ASS group, and positive non-Jo-1 antibodies were a significant independent predictor of PPF. Our data support that ASS-ILD patients with positive non-Jo-1 antibodies may have a potentially higher risk of developing PPF.

DLCO%pred and the PaO_2_/FiO_2_ ratio are used to evaluate the severity of lung impairment and predict prognosis in IPF patients [32]. In our cohort, DLCO%pred and the PaO_2_/FiO_2_ ratio were lower in the PPF-ASS group than those in the non-PPF-ASS group. Furthermore, univariate analysis revealed that DLCO%pred was a risk factor for PPF in patients with ASS-ILD. In turn, multivariate analysis demonstrated that DLCO was not an independent risk factor for PPF in these patients. In contrast to this observation, a previous study showed that lower baseline DLCO%pred predicted poor prognosis in 107 PM-DM patients with ILD [33]. In addition, Chen et al. [34] found that lower baseline of PaO_2_/FiO_2_ and DLCO%pred predicted poor prognosis in 72 patients with DM-ILD. This discrepancy in the results might be caused by differences in sample size, which warrants further investigation.

The NLR is a biomarker of inflammatory status [35]. Inflammatory cells release pro-fibrotic cytokines, chemokines, and growth factors that activate fibroblasts and stimulate inflammatory and tissue-remodeling pathways [36]. Therefore, elevated NLR may reflect a pro-inflammatory and pro-fibrotic status. Previous studies revealed that NLR ≥ 6.11 tended to be associated with poor prognosis in IIM patients, and NLR (95% CI: 1.019–1.056) might be a useful prognostic biomarker [37, 38]. In this study, NLR was elevated in the PPF-ASS group. Multivariate analysis demonstrated that NLR > 4.05 was an independent risk factor for PPF in these patients. These results imply that inflammatory and pro-fibrotic pathways are critical molecular mechanisms for PPF, and NLR may be a predictive factor for PPF development.

Consistent with previous studies [18, 39], our results suggest that the most common radiological patterns in our cohort were NSIP and NSIP/OP overlap. In contrast, UIP patterns and unclassifiable patterns are less observed. Reticular opacities were more common in the PPF-ASS group than in the non-PPF-ASS group and were a risk factor for PPF in patients with ASS-ILD but not an independent risk factor. Consistent with these observations, a previous study demonstrated that patients with NSIP did not respond to therapy and developed fibrosis. Park et al. [40] reported that a subset of patients with NSIP did not respond to therapy with a progressively deteriorating rate of 19% and disease-related mortality of 30%, similar to that in IPF. Cho et al. [41] observed that among 197 patients with biopsy-confirmed NSIP who received treatments, there was a disease progression in 71 (36%) patients during follow-up, and progression was the sole predictor of mortality. This result indicated that NSIP was common in patients with progressive interstitial fibrosis.

In this study, the initial treatment of CS monotherapy was higher in the PPF-ASS group than in the non-PPF-ASS group. Moreover, CS monotherapy was a predictor of PPF in patients with ASS-ILD but not an independent risk factor. CS with/without immunosuppressant therapy is the first-line treatment for patients with ASS-ILD. In actual clinical practice, some experts may administer high-dose CS monotherapy for patients with ASS-ILD as the first-line treatment at onset, this initial high-dose of CS monotherapy is usually maintained for 4 weeks, and immunosuppressants are started when the CS therapy is tapered. Some experts may use low-dose CS combined with immunosuppressive agents at the onset for patients with ASS-ILD. The treatment plan can be adjusted according to the follow-up results and patients are usually prescribed additional immunosuppressants after relapse [42]. Our results suggest that a combination therapy of CS and immunosuppressants may be superior to CS monotherapy alone, especially the combination therapy with immunosuppressants better be selected at onset. However, further comparative studies involving the different treatment strategies are needed to confirm our results.

In the study, poorer survival was observed in the PPF-ASS group, which was in line with previous studies. Chiu et al. [6] retrospectively evaluated 150 patients with CTD-ILD (RA, 16%; IIM, 19.3%) and observed that PF-ILD occurred in 76 (RA, 50%; IIM, 45%) patients. Furthermore, survival was worse in the PF-ILD group than that in the non-PF-ILD group, and 2-year overall survival was 80%. Conversely, the overall survival (median, 37.4 months) for PPF in our cohort was higher than previously reported. Considering that the proportion of patients with IIM in the previous study was small (19.3%), differences in survival might be due to differences in patient groups according to ILD subtypes. The prognosis of PPF was poor in our cohort.

The factors that best predicted PPF were identified by ROC analysis. Positive non-Jo-1 antibodies, NLR, and serum KL-6 were independent risk factors for PPF. The combined predictive value of these factors was the highest (AUC = 0.874), and sensitivity and specificity were 94.4% and 64.8%, respectively. Therefore, monitoring these markers can potentially predict PPF in patients with ASS-ILD.

Our study has several limitations. First, it included a relatively small sample size due to the relative rarity of ASS-ILD, which made it difficult to draw definitive conclusions from the multivariate analysis; hence, the results of the multivariate analysis must be interpreted with caution. Second, anti-KS, anti-OJ, anti-Zo, and anti-Ha antibodies were not detected because of their rarity. Third, the lack of risk factors changes over time. Fourth, some physiological parameters were missing from the follow-up data, and the follow-up period was short (median, 37.4 months). Despite these limitations, our study revealed that monitoring the levels of positive non-Jo-1 antibodies, NLR, and serum KL-6 might aid to predict PPF in patients with ASS-ILD. However, further larger multicenter studies with longer follow-up are needed to determine the best predictor on changes over time for PPF in ASS-ILD patients.

In conclusion, our study delineated risk factors of PPF in patients with ASS-ILD. Multivariate analysis revealed that positive non-Jo-1 antibodies, NLR, and serum KL-6 were independent risk factors for PPF in patients with ASS-ILD. Thus, the combined use of these indexes can potentially improve the prediction of PPF in these patients.

## Supplementary Information

Below is the link to the electronic supplementary material.

Supplementary file1 Supplementary Table S1. ROC analysis of the predictive value of isolated index for PPF in patients with ASS-ILD. ASS-ILD: anti-synthetase syndrome-associated interstitial lung disease; AUC: area under the ROC curve; KL-6: Krebs von den Lungen-6; NLR: neutrophil-to-lymphocyte ratio; PPF: progressive pulmonary fibrosis; ROC: receiver operating characteristic. Supplementary Table S2. ROC analysis of the predictive value of combination of risk factors for PPF in patients with ASS-ILD. ASS-ILD: anti-synthetase syndrome-associated interstitial lung disease; AUC: area under the ROC curve; KL-6: Krebs von den Lungen-6; NLR: neutrophil-to-lymphocyte ratio; PPF: progressive pulmonary fibrosis; ROC: receiver operating characteristic. Supplementary Fig. S1. ROC curve for analysis of the predictive value of the NLR to predict PPF. The optimal cutoff for the NLR to predict progressive pulmonary fibrosis was 4.05, the area under the curve was 0.758, sensitivity was 72.2%, and specificity was 74.1%. Supplementary Fig. S2. ROC curve for analysis of the predictive value of the KL-6 to predict PPF. The optimal cutoff for KL-6 to predict PPF was 644.71 U/mL, the area under the curve was 0.702, sensitivity was 94.4%, and specificity was 46.3%. Supplementary Fig. S3. ROC curve for analysis of the predictive value of the combination of risk factors for PPF (PPTX 205 KB)

## Data Availability

The data that support the findings of this study are available on reasonable request from the corresponding author.
