# Correction to: Prediction of progressive pulmonary fibrosis in patients with anti-synthetase syndrome-associated interstitial lung disease

**DOI:** 10.1007/s10067-023-06583-y

**Published:** 2023-03-27

**Authors:** Hongyan Fu, Ziyao Zheng, Zhenping Zhang, Yanjuan Yang, Jieda Cui, Zhaojun Wang, Jing Xue, Shuhong Chi, Mengshu Cao, Juan Chen

**Affiliations:** 1 Department of Key Laboratory of Ningxia Stem Cell and Regenerative Medicine, Institute of Medical Sciences, General Hospital of Ningxia Medical University, Yinchuan, 750004 Ningxia China; 2 Department of Pulmonary and Critical Care Medicine, General Hospital of Ningxia Medical University, Yinchuan, 750004 Ningxia China; 3 Ningxia Medical University, Yinchuan, 750004 Ningxia China; 4 Department of Radiology, General Hospital of Ningxia Medical University, Yinchuan,, 750004 China; 5 Department of Pulmonary and Critical Care Medicine, General Hospital of Xinmi Traditional Chinese Medicine, Xinmi, 452370 Henan China; 6 Institute of Human Stem Cell Research, General Hospital of Ningxia Medical University, Yinchuan, 750004 Ningxia China; 7 Department of Rheumatology, General Hospital of Ningxia Medical University, Yinchuan, 750004 Ningxia China; 8 Department of Respiratory and Critical Care Medicine, The Affiliated Drum Tower Hospital of Nanjing University Medical School, No. 321 Zhongshan Road, Nanjing, 210008 Jiangsu China; 9 Department of Critical Care Medicine, General Hospital of Ningxia Medical University, Yinchuan, 750004 Ningxia China

**Correction to: Clinical Rheumatology**

10.1007/s10067-023-06570-3

The attributes of the affiliations for the authors Ziyao Zheng, Zhenping Zhang, Zhaojun Wang, Jing Xue, Shuhong Chi, Mengshu Cao were incorrect and has been corrected as follows:

**Ziyao Zheng** ^**2,5**^

2 Department of Pulmonary and Critical Care Medicine, General Hospital of Ningxia Medical University, Yinchuan 750004, Ningxia, China

5 Department of Pulmonary and Critical Care Medicine, General Hospital of Xinmi Traditional Chinese Medicine, Xinmi 452370, Henan, China

**Zhenping Zhang** ^**4**^

4 Department of Radiology, General Hospital of Ningxia Medical University, Yinchuan 750004, China

**Zhaojun Wang** ^**2,9**^

2 Department of Pulmonary and Critical Care Medicine, General Hospital of Ningxia Medical University, Yinchuan 750004, Ningxia, China

9 Department of Critical Care Medicine, General Hospital of Ningxia Medical University, Yinchuan 750004, Ningxia, China

**Jing Xue** ^**6**^

6 Institute of Human Stem Cell Research, General Hospital of Ningxia Medical University, Yinchuan 750004, Ningxia, China

**Shuhong Ch**i^7^

7 Department of Rheumatology, General Hospital of Ningxia Medical University, Yinchuan 750004, Ningxia, China

**Mengshu Cao** ^**8**^

8 Department of Respiratory and Critical Care Medicine, The Afliated Drum Tower Hospital of Nanjing University Medical School, No. 321 Zhongshan Road, Nanjing 210008, Jiangsu, China

The original article has been corrected.

