# Supplementary material for: Prediction of progressive pulmonary fibrosis in patients with anti-synthetase syndrome-associated interstitial lung disease
Source: Clin Rheumatol. 2023 Mar 17;42(7):1917–29. doi: 10.1007/s10067-023-06570-3 (PMC10266998; doi:10.1007/s10067-023-06570-3)
Supplement: Supplementary file 1 — Supplementary file1 Supplementary Table S1. ROC analysis of the predictive value of isolated index for PPF in patients with ASS-ILD. ASS-ILD: anti-synthetase syndrome-associated interstitial lung disease; AUC: area under the ROC curve; KL-6: Krebs von den Lungen-6; NLR: neutrophil-to-lymphocyte ratio; PPF: progressive pulmonary fibrosis; ROC: receiver operating characteristic. Supplementary Table S2. ROC analysis of the predictive value of combination of risk factors for PPF in patients with ASS-ILD. ASS-ILD: anti-synthetase syndrome-associated interstitial lung disease; AUC: area under the ROC curve; KL-6: Krebs von den Lungen-6; NLR: neutrophil-to-lymphocyte ratio; PPF: progressive pulmonary fibrosis; ROC: receiver operating characteristic. Supplementary Fig. S1. ROC curve for analysis of the predictive value of the NLR to predict PPF. The optimal cutoff for the NLR to predict progressive pulmonary fibrosis was 4.05, the area under the curve was 0.758, sensitivity was 72.2%, and specificity was 74.1%. Supplementary Fig. S2. ROC curve for analysis of the predictive value of the KL-6 to predict PPF. The optimal cutoff for KL-6 to predict PPF was 644.71 U/mL, the area under the curve was 0.702, sensitivity was 94.4%, and specificity was 46.3%. Supplementary Fig. S3. ROC curve for analysis of the predictive value of the combination of risk factors for PPF (PPTX 205 KB) [file 10067_2023_6570_MOESM1_ESM.pptx]

## Slide 1
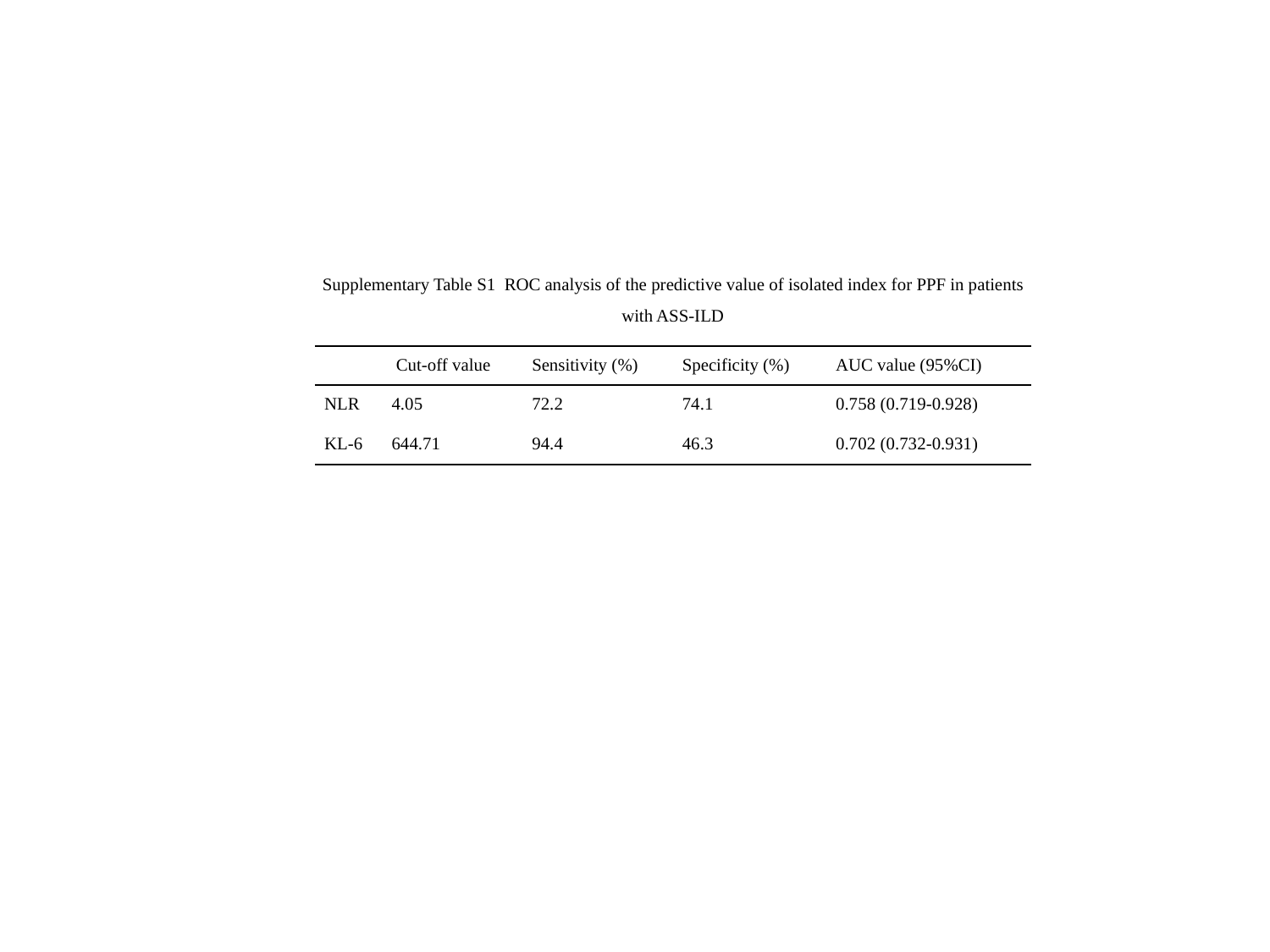

Supplementary Table S1 ROC analysis of the predictive value of isolated index for PPF in patients with ASS-ILD
| | Cut-off value | Sensitivity (%) | Specificity (%) | AUC value (95%CI) |
| --- | --- | --- | --- | --- |
| NLR | 4.05 | 72.2 | 74.1 | 0.758 (0.719-0.928) |
| KL-6 | 644.71 | 94.4 | 46.3 | 0.702 (0.732-0.931) |

## Slide 2
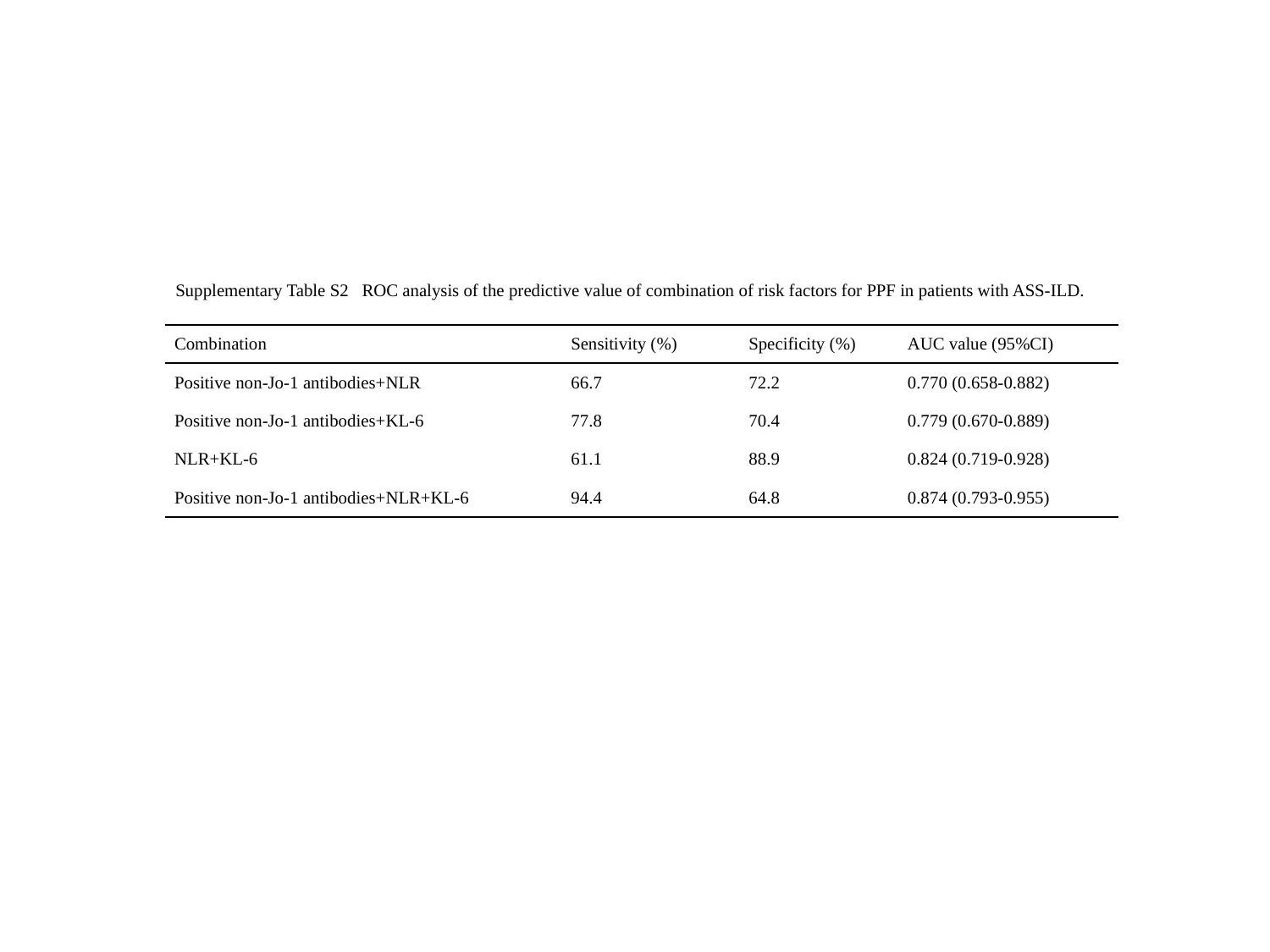

Supplementary Table S2 ROC analysis of the predictive value of combination of risk factors for PPF in patients with ASS-ILD.
| Combination | Sensitivity (%) | Specificity (%) | AUC value (95%CI) |
| --- | --- | --- | --- |
| Positive non-Jo-1 antibodies+NLR | 66.7 | 72.2 | 0.770 (0.658-0.882) |
| Positive non-Jo-1 antibodies+KL-6 | 77.8 | 70.4 | 0.779 (0.670-0.889) |
| NLR+KL-6 | 61.1 | 88.9 | 0.824 (0.719-0.928) |
| Positive non-Jo-1 antibodies+NLR+KL-6 | 94.4 | 64.8 | 0.874 (0.793-0.955) |

## Slide 3
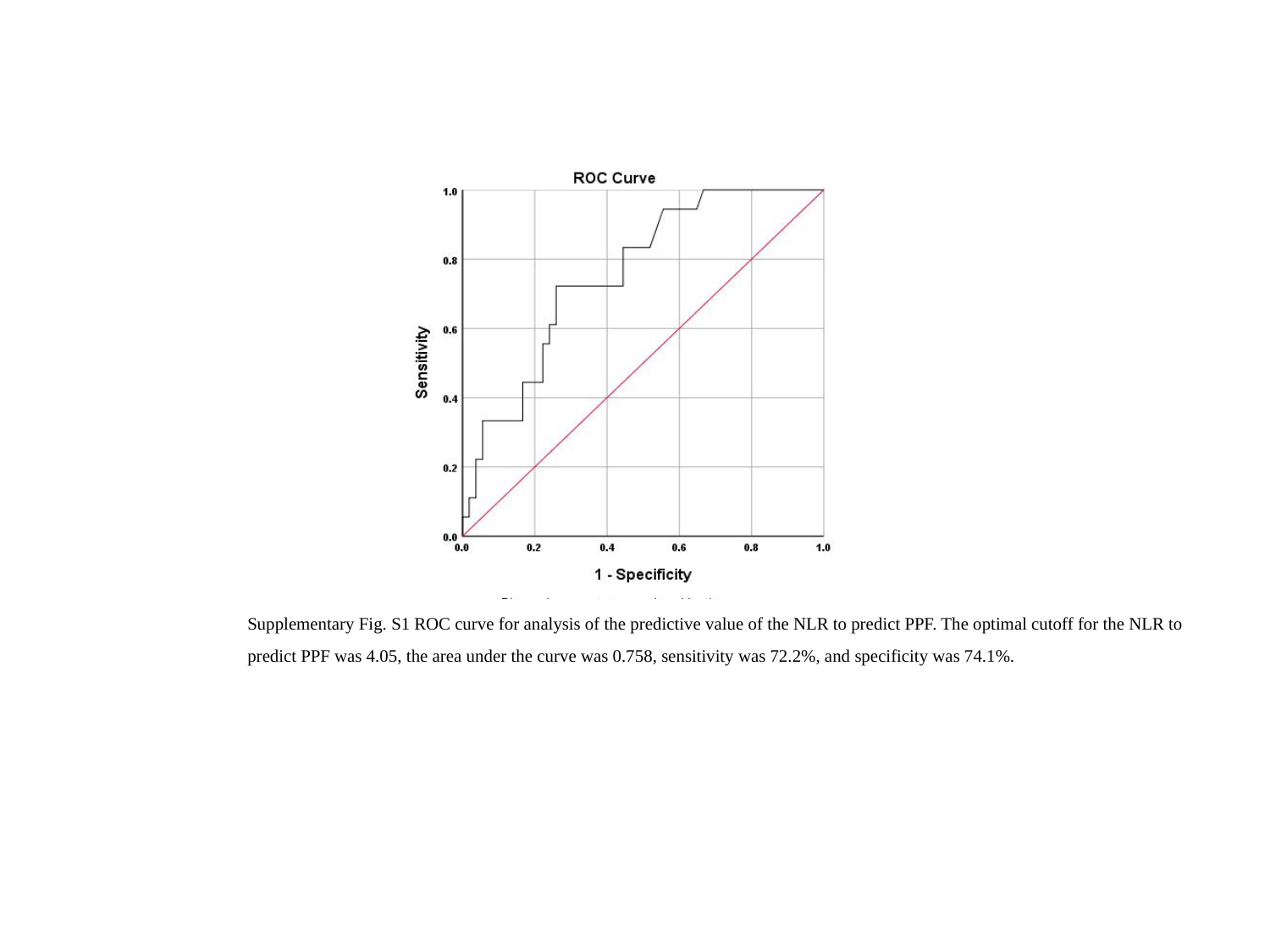

Supplementary Fig. S1 ROC curve for analysis of the predictive value of the NLR to predict PPF. The optimal cutoff for the NLR to predict PPF was 4.05, the area under the curve was 0.758, sensitivity was 72.2%, and specificity was 74.1%.

## Slide 4
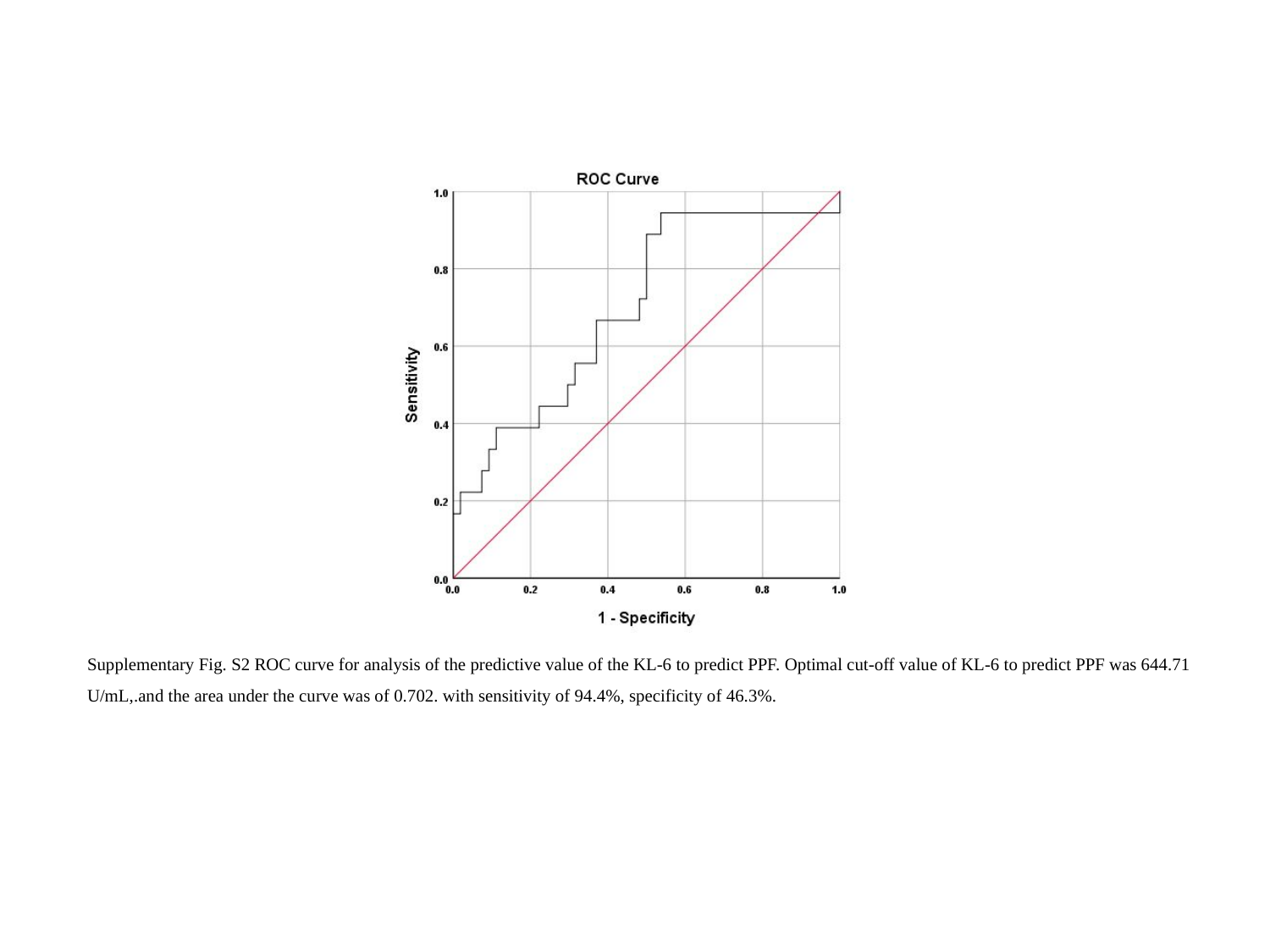

Supplementary Fig. S2 ROC curve for analysis of the predictive value of the KL-6 to predict PPF. Optimal cut-off value of KL-6 to predict PPF was 644.71 U/mL,.and the area under the curve was of 0.702. with sensitivity of 94.4%, specificity of 46.3%.

## Slide 5
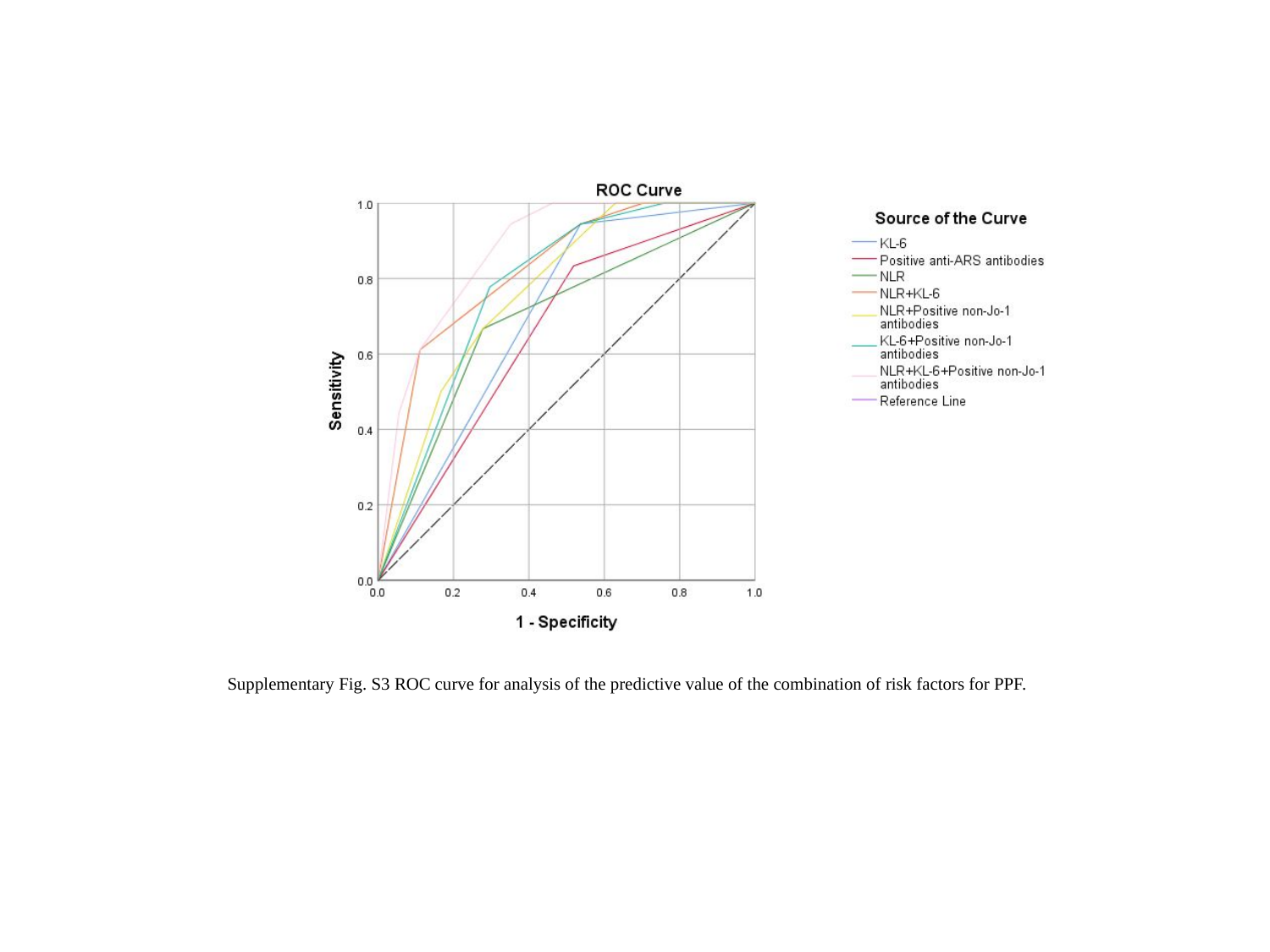

Supplementary Fig. S3 ROC curve for analysis of the predictive value of the combination of risk factors for PPF.
